# Exploring the Functioning of Decision Space: A Review of the Available Health Systems Literature

**DOI:** 10.15171/ijhpm.2017.26

**Published:** 2017-02-27

**Authors:** Tamlyn Eslie Roman, Susan Cleary, Diane McIntyre

**Affiliations:** Health Economics Unit, University of Cape Town, Cape Town, South Africa.

**Keywords:** Decision Space, Health System, Review

## Abstract

**Background:** The concept of decision space holds appeal as an approach to disaggregating the elements that may influence decision-making in decentralized systems. This narrative review aims to explore the functioning of decision space and the factors that influence decision space.

**Methods:** A narrative review of the literature was conducted with searches of online databases and academic journals including PubMed Central, Emerald, Wiley, Science Direct, JSTOR, and Sage. The articles were included in the review based on the criteria that they provided insight into the functioning of decision space either through the explicit application of or reference to decision space, or implicitly through discussion of decision-making related to organizational capacity or accountability mechanisms.

**Results:** The articles included in the review encompass literature related to decentralisation, management and decision space. The majority of the studies utilise qualitative methodologies to assess accountability mechanisms, organisational capacities such as finance, human resources and management, and the extent of decision space. Of the 138 articles retrieved, 76 articles were included in the final review.

**Conclusion:** The literature supports Bossert’s conceptualization of decision space as being related to organizational capacities and accountability mechanisms. These functions influence the decision space available within decentralized systems. The exact relationship between decision space and financial and human resource capacities needs to be explored in greater detail to determine the potential influence on system functioning.

## Background

Decentralization is offered as a key reform in strengthening and improving health system functioning and effectiveness. It describes a process of change which could result in a range of bureaucratic outcomes along a continuum involving multiple decision-making mechanisms for control over various functions.^1-4^ Yet, in its most basic conceptualization, it can be defined as a transfer of decision-making authority from the centre to the periphery.^5,6^

Within this context, the idea of decision space^7,8^ which was first conceptualized by Bossert presents one approach to aid in the understanding of how decentralization is operationalized within health systems, by defining the degree of choice at local levels and the transfer of decision-making capacity in decentralized organisations.^1,9-12^

The decision space approach offers the opportunity to assess the degree of decentralization granted for different health system functions and allows for the disaggregation of the extent of the transfer of authority for decision-making from the centre to the periphery. According to Faguet this is a more realistic way of assessing real-world experience than merely considering a dichotomous centralized-decentralized outcome.^5^

Formally, decision space is defined by how much authority for making decisions on different functions is delegated to local authorities from above through official policies. However, in reality, local authorities may exercise a different degree of choice, which Bossert and Mitchell refer to as their informal or *de facto* decision space.^9^

Many assessments of decentralization consider who is given more authority over decision-making but less attention is paid to what that authority entails. In adopting a functional approach to the conceptualization of decision space, Bossert and Mitchell address this shortcoming and identify organizational capacities and accountability mechanisms as being important to the exercise of decision space and the definition of what range of choice is allowed.^8,9^ Their conceptualization of decision space as being related to organizational capacity and accountability mechanisms provides a very useful framework for assessment.

This review utilizes Bossert and Mitchell’s framework as a starting point in order to further explore the factors that influence de facto decision space within decentralized systems. By reviewing the existing empirical literature, this article seeks to explore in more details the functioning of decision space and the factors which facilitate or impede decision space – particularly organizational capacities and accountability mechanisms.

## Methods

### Data Search Strategy and Selection Criteria

We searched for relevant literature during January 2015, repeating the search in July 2016, in PubMed Central, Science Direct, Wiley, Sage, Emerald and JSTOR using the search string *“(decision space OR management decision OR management autonomy) AND (organizational capacity OR resources OR decentralization) AND (health system).*”

The initial work conceptualizing the impact and functioning of ‘decision space’ in the health system was published in 1998.^7^ Under the assumption that empirical work related to this notion would likely be published from 2000 onwards, the search was limited to studies published between January 2000 and July 2016. The titles and abstracts of papers matching these search terms were reviewed and only those related to the health system were considered for final inclusion. Only articles that had undergone peer review through reputable journal sources were included in an effort to ensure the quality of the source of data. Only papers written in English and available online, in full text, were included in the review.

The focus of this article is on the functioning of decision space – particularly at the organizational level because this presents a bounded space in which to review the interplay between decision space, accountability and organizational capacity. Organizations can exist at various levels of the health system and can vary in size, structure and intended service delivery outcomes – from hospitals and health facilities to district and national level authorities. Thus, the articles included in the review encompass a broad scope of insights regarding decision space in the functioning of organizations across the health system.

As illustrated in Figure , the initial search yielded 925 papers after removing duplicates from searches conducted across the different search engines. On the basis of the title, 787 articles were excluded because they were not related to health systems. The remaining 138 articles were downloaded in full and were screened based on the criterion that they provided insight into the functioning of decision space either through the explicit application of or reference to decision space, or implicitly through discussion of decision-making related to organizational functioning. Inclusion of these articles was based on their ability to shed light specifically on the relationships between organizational capacities, accountability mechanisms and decision space. The articles that were excluded did not explicitly acknowledge decision space nor did they refer to any concept or practice related to how decisions are made, the scope of decision-making or what the outcomes of decision-making might be. Seventy-six articles were finally included in the review.

**Figure:**
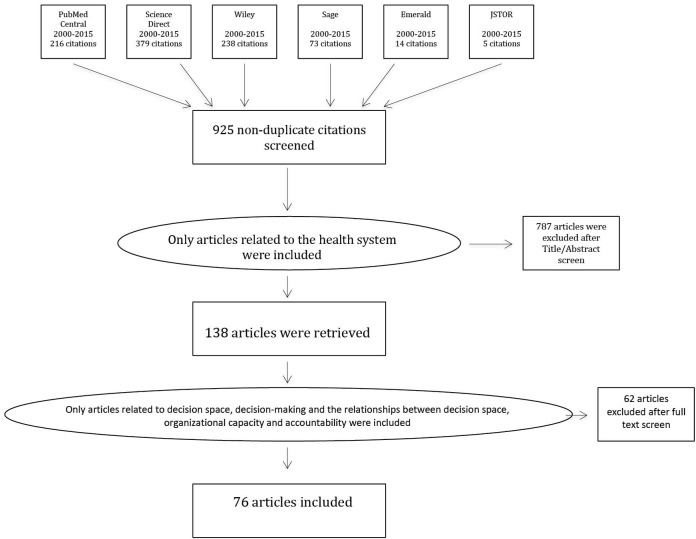
Results Flow Diagram.

The search results and articles included in the review have been summarized in Table . More than half of the papers looked at management practices or outcomes within the health sector, primarily at the institutional or district level, and 31 papers considered the outcomes or implementation of decentralization. The majority of the articles contributed to an understanding of financial or management organizational capacities. Most were case studies from African and Asian settings. Far fewer articles looked at European or Latin American settings and no studies were from North America. One study was based in Fiji and four review papers presented comment on it that contributed to the four articles based in the Oceania region. Data collected were both quantitative and qualitative and methodologies included document reviews, in-depth interviews and surveys.

**Table:** Frequency of Articles by Category

**Study Focus/Method/Region**	**Frequency**
Decision Space	17
Decentralization	41
Management	49
Qualitative	59
Quantitative	7
Review	12
Africa	24
Asia	23
Europe	6
South America	4
North America	0
Oceania	4
Organizational capacity (finance)	25
Organizational capacity (human resources)	18
Organizational capacity (management)	28
Accountability	21
Context	20

All of the papers included either referenced the term decision space or one of the Bossert papers on decision space.^1,7,9^ However, only 17 of the papers actually applied the decision space approach or considered the decision space available to managers in the study setting.

### Data Extraction

Data extraction took into consideration Bossert and Mitchell’s framework, which was then used as a basis for manually processing the articles to identify the relevant contributions. This review does not attempt to determine a quantitative outcome but rather to explore the conceptual foundations of decision space, thus no risk of bias assessment was made and a quality consideration of the articles included would not have an impact on the thematic findings.

### Data Analysis

Publications were reviewed for information regarding the determinants of decision space, and the influence of factors such as organizational capacity and accountability on the functioning of decision space. Specific attention was paid to the application of these concepts to decentralized health systems, in general, and organizational functioning, in particular.

## Results

The themes which emerged as a result of the literature review suggest that the decision space available for managers to utilise is influenced by the resources that they have at their disposal: their own management capacities; the accountability mechanisms to which they are subjected; and the context in which they operate.^10^

These articles provide conceptual clarity on the functioning of decision space with most studies focusing on the influence of one specific functional area rather than all of the articles addressing all of the functional areas. For example, Atkinson et al do not explicitly examine the relationships between decision space, accountability mechanisms and capacities but they do complement the decision space framework by exploring the influence context has over service provision in further detail.^13^ However, some of the articles consider more than one of the functional areas – such as Marchal and Kegels who approach their assessment of decision space with a greater emphasis on management and human resource capacities.^14^ The articles that had a more multi-functional, systems orientation offered more utility for the development of an understanding of how decision space functions and how it is defined.

The results section is divided into the findings related to decision space, organizational capacity and accountability mechanisms. For each of these functional areas, a brief overview of the concept is provided before it is assessed in terms of the relationship with decision space functioning – the ability of local officials to make and implement decisions that are likely to improve effectiveness, efficiency and quality of care. Lastly, the contribution of context is considered.

Decision space, and the relationship between decision space and organisational capacities and accountability respectively are discussed in the sections that follow.

### Defining Decision Space

Decision space is a term used to describe the range of choice, or authority and responsibility, which decentralized organizations have been granted by central authorities to make decisions about or influence a range of functions and resources. It is characterized by both formal and informal range of choice.^7^

Decision space represents the degree of decentralisation granted to an individual or an organisation and the assumption is that with increased decision space, managers could make decisions that are more innovative, efficient and responsive to local conditions and that this would improve the quality of service delivery.^5,9^

Increased decision space is thus not an end in itself, but rather is a management or organisational approach that aspires to enable performance improvements. It exists, theoretically, on a continuum ranging from none^5,11,15^ to complete decision space with a multitude of variations across functional areas.

Formally, decision space is determined by the institutional arrangement that grants authority.^10^ According to Mills et al, “health systems can be described in terms of the relative roles and responsibilities given to the different levels of the health system, from the national government at the top, down to the individual facility level.”^16^ The roles assumed by the various institutions at different levels of the system are determined by the degree of decentralization and the distribution of authority. Thus, within each organizational system, the responsibilities assumed and the associated decision space available, are unique, and fall anywhere across a wide spectrum of options.^17-19^

Decentralization requires a change in actors’ and institutions’ roles and responsibilities within the organizational structure and this changes the degree of decision space available.^20^ Yet a recurring concern identified in the literature is that of unclear boundaries between central and local authorities and lack of clarity regarding who gives instructions, who has jurisdiction and who is responsible for implementation.^16,21-25^ Having unclear responsibilities and multiple authorities could lead to confusion regarding the available decision space.

In line with Bossert’s proposal of formal and informal decision space, while the allocation of authority is formally determined by legislation, other *de facto* factors – such as the influence of other functional areas of the health system – may influence the actual roles in practice.^10,16^ Therefore, in defining the decision space, there is a need to consider not just the formal policies and legislation mandating authority but also the various processes and relationships which may influence the actual range of choice afforded to local authorities and, thus, how the organization actually functions.^1,8,16,25^

### Applications of Decision Space

Overall the decision space literature supports the idea that constraining the extent to which authorities have real power to influence decision-making related to some of the functional areas adversely impacts on the attainment of the full theoretical benefit of decentralization. In other words, with little change in the decision space available to local authorities, the rhetoric of decentralization is not always realized in implementation.

The decision space approach has been applied to case studies in Pakistan,^6,9^ Bolivia,^8^ Chile,^8^ India,^8,26^ the Phillipines,^8^ Uganda,^8^ Fiji,^11^ Ghana,^10,12,27^ South Africa,^28^ Vietnam,^29^ and Tanzania.^30^

Most of these studies considered the extent of authority local managers have over health system functions following the introduction of decentralization reforms. For example, Kwamie et al conducted a study considering the available decision space in Ghana over time. They found that shortly after the implementation of a decentralized district health system, district manager decision space increased due to efforts to increase management capacity. However, decision space has since decreased with little change in the system of centralized decision-making.^12^

In Fiji, Mohammed et al found that despite being granted *de jure* decision space in terms of legislation supporting decentralization, local managers’ *de facto* decision space was close to zero. This supports the idea that the possible positive benefits of decentralization are dependent on local managers being enabled to exercise decision space.^11,26^

### Decision Space, Organizational Capacity and Accountability Mechanisms

All of the decision space articles, in applying a framework of assessment that considers the degree of decision space as well as the concomitant organizational capacity and accountability mechanisms, contribute to a better understanding of how decision space functions.

For example, in Ghana, Kwamie et al found that resource uncertainty decreased decision space and Marchal et al found that merely having decision space did not guarantee good management but rather that decision space had to be accompanied by management capabilities and leadership skills.^10,12,31^ In India, the perception of limited decision space led to limited community participation in planning and priority setting and thus, less responsive outcomes, and similarly to the Ghanaian studies, that local managers who are capacitated to function more autonomously are better equipped to improve health systems performance.^26^

However, few studies have explicitly explored the interactions between decision space, organizational capacity and accountability mechanisms and how these latter two functions influence the functional decision space available.

Bossert and Mitchell investigated the relationship between the dimensions of decentralization – decision space, organizational capacity and accountability mechanisms – in Pakistan.^9^ They did not find evidence of a relationship between decision space and accountability. However, they did find a strong positive correlation between organizational capacities and accountability and between organizational capacities and decision space.^9^ This study formed the foundation for the authors’ second decision space study in Pakistan which attempted to assess the impact of capacity building and changes in decision space on performance.^6^ Again, they identified “synergistic relationships” suggesting that increases in organizational capacity may be accompanied by increases to decision space, which, if supported by greater accountability to local officials, may improve health system performance.^6^

While the above is evidence of some of the work that has been done to assess the functioning of decision space, there are still a limited number of studies that focus explicitly on the relationship between decision space, organizational capacities and accountability mechanisms. This review has identified literature that aids in the development of this conceptualization.

The results that follow explore the literature regarding organizational capacity and accountability mechanisms *in relation to decision space*.

### Defining Organizational Capacities

According to the United Nations Development Programme, *organizational capacities* are defined as “the ability of individuals, organizations or systems to perform appropriate functions effectively, efficiently and sustainably.”^9^ Within an organization, these capacities involve administrative, technical, organizational, financial, and human resources.^9^

Traditionally, the primary resource inputs into organizational functioning are financial and human resources.^8,9^ These resources capacitate functioning and they influence the extent to which local authorities – whether they are lower levels of government or individual public facilities or entities – are capable of executing their tasks.

Given the specificity of the articles reviewed, many of the studies considered capacities in the context of decentralisation – assessing whether decentralisation yielded any change in the availability of resources or whether the availability, or lack, of these resources had any influence on the outcomes of decentralization policies.^2-4,10,16,17,21,23,24,29,30,32-35^

The literature widely acknowledges the importance of resource capacities as inputs into organisational functioning. Yet there is no evidence that has shown that a high level of resources is able to have a direct impact on the effectiveness and efficiency of services. This is because resource inputs alone are an insufficient determinant of functioning. It is also necessary that they be managed effectively.

However, again, in an under resourced system, it is a challenge to determine to what extent additional management capacities could alter the outcome. The literature does not always acknowledge the interdependence between the availability of resource capacities and their management^16,23,31,32^; nor does it always differentiate between the influences of the different forms of capacity.^14,21,36-38^

For the purposes of this review, financial and human resource input capacities refer to the availability of resources, and reference to management capacities encompasses their management as well as the management of the organization as a whole.

One shortcoming of this review is that the organizational capacities that have been given attention are limited to financial, human resource and management capacities. Additional capacities such as infrastructure or information systems were not explored in as much depth – largely because they did not emerge as a strong focus of the articles that were retrieved during the literature search.

### Organisational Capacity and Decision Space Functioning

Based on the definition of organization capacity discussed previously, the consideration of organizational capacity’s influence on decision space functionality has been disaggregated into financial resource, human resource and management capacities.

The articles that were most useful to the aim of this review were the ones that considered multiple capacities or functions within one system and identified relationship between them. Studies that had a very specific focal point, though relevant, did not assist as significantly to the synthesis of possible explanations of decision space functioning.

#### Financial Resource Capacity and Decision Space Functioning

Financial resource capacity is important to the functioning of any organization as it enables greater scope for action. It is the outcome of the availability of funding and the authority to make use of and allocate those funds.^8^

The influence that money has over the functioning of the system is granted to whoever controls the financing sources, funding flows and budget allocations and the degree of control over these finance components therefore influences the degree of available decision space. With access to greater financial resources comes greater responsibility and therefore, theoretically, more decision space.

However, Munga et al argue that there is a recurring pattern of local authorities being assigned increased responsibilities without being capacitated through increased financial resources to act on them.^4,13,20,39^ “Districts are being assigned too many responsibilities that do not match with the resources at their disposal.”^34^ They refer to this as “responsibility without resources and authority.”^34^

For example, in some systems, decision-making over budget allocations has been decentralized to the local level but central government still controls the funding flows and allocations to the local level. Thus, while decentralization policies grant managers more theoretical authority over budgetary resources, their actual financial resource capacity and their ability to make decisions regarding financing is very rarely changed.^11,21,35,38,40-42^

In the study conducted by Asante et al on the routine availability of financial resources for district health services in Ghana, one director observed that, “The timing is bad; I mean bad, really bad! They are not regular at all…we understand that the budget has to go through some process, say from the district to the region, to national, then it will go to the Ministry of Finance and probably Parliament has to approve it before the money can be released. But that is no excuse, something has to be done about it; they should find a way to solve it. Sometimes you stay up to June and nothing has come, meanwhile that is half the year gone so what services are you going to render and with what? Sometimes it is so demoralizing you just don’t know what to do.”^38^

Centrally controlled financing is a problem when allocations do not meet the need, or when funding is delayed because it prevents programmes being implemented according to plans made locally.^30,38,40,43^

According to Ensor et al, “being able to deliver on responsibilities implies that local bodies are able to control the use of the budgets they are allocated. Yet the continued use of centrally operated allocation systems, sometimes supplemented by new restrictions, can mean that this flexibility is severely curtailed.”^20^

Centralized control narrows the available decision space for local level managers.^44^ To counter the uncertainty faced by haphazard resource flows, managers may resort to informal strategies to compensate for the resource limitations. They may rely on the relationships they have within the system and on informal strategies – such as saving money to use at their discretion.^44^ This reflects what Bossert refers to as the “informal decision space”^7^ whereby local officials “bend the rules” to challenge the degree of decision space granted to them. It is possible that the informal decision space is utilized more when the formal decision space inhibits fulfilling the organization’s management and service delivery mandates.^10^

The challenge of continued centralized control is that it perpetuates the administrative delays which decentralization is supposed to address.^45^ Increased decision space over financial resources could therefore influence the speed with which actions are taken, and also make service delivery more responsive to local conditions.

Similar limitations on decision space are apparent in cases where health facility level managers are given control over areas such as cost recovery and patient fee setting but are not granted control over how these finances are to be spent. In this instance, the supervision and control exerted by the local authorities and the Ministry of Health have a limiting influence on managers’ decision space.^46^

It is also important for financial resource capacities to align with local conditions. Policies may permit health facility managers increased autonomy to mobilize financial resources and to determine how best to utilize those resources. However, this new delegation will become redundant if the sources of local revenue are limited. The health facility will then remain dependent on central government financing and allocation decisions.^30,47^

#### Human Resource Capacity and Decision Space Functioning

The second major input that capacitates decision-making is human resources. As with financial resources, human resource capacity is derived from both the availability of workers but also the authority to make decisions regarding their management.^8^

Some of the main concerns related to decision space and human resource management involve administrative delays during recruitment, the appropriateness of postings and retention of staff.^9,34,48-51^ Increased decision space, theoretically, could address some of these challenges by granting local managers greater flexibility over human resource management.^7,51^

The recruitment process is often lengthy and complex. There are set procedures that need to be followed to make the process fair, and these, in addition to time consuming administrative delays – which result from the bureaucracy which accompanies centralized systems – mean that it can take a long time for vacancies or new posts to be filled. Furthermore, these delays are often exacerbated by a lack of qualified applicants.^34^

With greater decision space for human resources management, managers could have the capacity to request additional placements for specific roles that they prioritize instead of the centralized bureaucracy independently making decisions that may not take the specific staffing needs of local facilities into consideration.^2^ In Tanzania, decentralization was credited for giving district authorities increased capacity to make requests based on the needs of the district, unlike under centralized management where they had little control over human resources postings.^34^

Yet, much like with financial resource capacities, the theoretical decision space available for human resource management should be matched by practical delegations which capacitate this in order for the change to be effective.^48^ For example, in Indonesia before decentralization, district-level managers had no control over hiring and firing of permanent staff but could create some flexibility in their skill mix through the hiring of contract staff. After decentralization, central government began to convert contact posts into permanent ones thereby reducing the available decision space for district managers.^21,49^

With regard to Munga colleagues’ “responsibilities without resources and authority,” “the authority to manage health personnel issues is constantly overridden by a number of central government organs. This leaves very little room for the district authorities to have a say in the management of their health workers and seriously reduces the effectiveness of the decentralized recruitment, retention and distribution of workers across districts.”^34^

Another component of human resource management is retention of staff. Maintaining a consistent team develops competencies, improves the team morale, minimizes disruptions in work systems, increases institutional knowledge and builds trust between the community and their service providers.^52^ It also assists in generating commitment to the objectives of the organization. Greater commitment may be found from those staff members who live in the community, or those who have worked in the organization on a long-term basis.^13^ Having a team that is committed and willing to work towards shared objectives means that the organizational capacity to successfully implement decisions is improved. Having the support of the organization better enables decision space, which in turn can be used to implement the organizational objectives.^14,21,29^

However, financial and human resource capacity alone is a necessary but insufficient condition for effective functioning of decision space; there also needs to be sound management.^37^

#### Management Capacity and Decision Space Functioning

Financial and human resource capacities relate to the organizational inputs required for functioning. As discussed, it is not only necessary for these resources to be available, but also for managers to have the authority to make decisions regarding their utilization. However, merely having the authority to make decisions is not sufficient. Management capacity is the competency to effectively utilize those resources to achieve the desired outcomes.

Decentralizing authority provides lower-level managers with greater decision space and in so doing, eliminates some of the bureaucratic constraints that exist within the system. It also gives them greater opportunity to make choices that are suitable for the local context.

However, being able to effectively utilize increased decision space is contingent upon a number of factors, namely that local managers have the capacity, knowledge and skill to develop and implement comprehensive plans.^30,36,51^ In other words, managers who are granted greater decision space over delivery of services are expected to have the competencies to make informed decisions and implement the decisions they make.^46,53^

In many developing country health systems, there is a need to develop management capacity for leadership, planning, resource allocation and financial management. According to Sherr, there is limited evidence on how best to do this, yet various suggestions have been made.^54^ Some of these include identification and prioritization of management challenges, regular planning and evaluation cycles, improved communication systems and training activities. The key capacities mentioned include the capacity for financial planning and management, human resource management, the establishment of a sound information management system and improved data utilization.^3,32,37,54-57^ With regard to the latter, in order to respond to local needs, the health system requires sufficient information as well as the capacity to plan and implement programmes in response to these needs. This is why it is vital to have health information systems that are both responsive and user-friendly.^58^ The other vital capacities are management of medicines, equipment and supplies and infrastructure development.^55,59^

However, management capacity requires more than decision-making competencies in the technical or functional areas of running a hospital. Managers are also required to make non-technical, leadership decisions which range from who to include in management decisions, and how to assign responsibility, to how best to create a motivational climate for staff and how to improve patient satisfaction.^60-63^

London found that where health facility management had greater decision space, managers appeared to have greater organisational commitment, a desire for continued learning related to management and finances, and ambitions to replicate successful models.^29^ Managers who are committed and who have the capacity to affect change have been shown to improve deliverables even under resource constraints.^64^

However, in a Tanzanian decentralization study, the district health management teams being granted greater responsibility felt that they lacked the capacity to adequately perform in the new roles.^20^ Having increased decision space could also easily lead to poor outcomes if management does not have the capacity to lead, to manage limited resources in such a way as to make them effective, and to negotiate the social and political context of the organization.^32^

### Defining Accountability Mechanisms

As a result of its complexity and multiple applications, the definition of accountability assumes a myriad of forms and constructs. However, at its core, accountability mechanisms hold decision-makers responsible for both doing the right thing and for doing it effectively.^8^ This can be operationalized to prevent abuses of power and to make decision-making more responsive to local needs.^9^

Accountability mechanisms can be defined as the assurances “of checks and restraints on power and discretion, of increased oversight and scrutiny, or of closer connections between service users and providers.”^65^ They are designed to make service delivery more responsive to local needs and, in ensuring that powers are not abused, to act as a counterbalance to full autonomy.

Examples of accountability mechanisms include hospital governing boards, financing mechanisms that link funding with performance; quality assurance policies that monitor standards and establish compliance mechanisms; human resource performance evaluations and key performance assessments; as well as health service and outcome targets.^66^

There are two kinds of accountability mechanisms: External and bureaucratic accountability – sometimes referred to as horizontal and vertical accountability respectively.^66^

External mechanisms aim to encourage accountability through community involvement in health facility governance.^66^ One of the key principles of many health systems is that “people have the right and duty to participate individually and collectively in the planning and implementation of their health care.”^67^ Part of the objective of increasing accountability in health is to ensure that the decisions being made are responsive to local needs, and that health systems are accountable to those people, or communities, that are involved in this process.

There are various mechanisms and platforms that can be used to support external accountability but the one that has received considerable attention within the literature on health facility accountability is the role of health facility governing boards – comprised of both health facility and community representatives. These boards have varying responsibilities ranging from strategic planning and budget approval to maintaining performance standards.^68^

Bureaucratic or internal accountability mechanisms, on the other hand, aim to establish accountability within the different levels of the health facility or health system – for example, between a health facility and central authorities.^66^ To make bureaucratic accountability mechanisms effective, there needs to be extensive monitoring and access to information.^63^

Of the articles assessed, only seven had accountability as the primary focus and of these the majority considered the community accountability mechanisms and the role of hospital governing boards.^19,58,66-70^ There was not an extensive assessment of the extent of accountability resulting from changes to or introductions of bureaucratic accountability mechanisms. Insights into the latter were derived from studies on governance and decentralization reforms.^16,20,21,41-43^ These articles suggest that the introduction of decentralization policies that were not sufficiently explicit in defining and respecting the lines of reporting and responsibility, led to weakened accountability within the system.^21,23,28,32,33,71,72^

What was noticeably lacking from the literature identified by the search was significant acknowledgement of the internal bureaucratic accountability mechanisms that a facility is subject to, for example, internal audits, target setting or supervision of facilities. These mechanisms, which often rely on centralised authority, play a role in setting organizational objectives, aligning local functioning with broader health system planning in decentralised systems and preventing the misuse of resources.^66^

### Accountability Mechanisms and Decision Space Functioning

In order to understand the degree of decision space granted to management, it is also important to explore the existing accountability mechanisms.

The nature of the accountability mechanisms to which a health facility is subject influences the degree of decision space a manager has but also assists in directing the kinds of decisions that can be made.

For example, the intention of having health facility boards is to encourage greater engagement with those communities that fall within a health facility’s catchment area. Unfortunately, evidence suggests that these mechanisms have been compromised by an insufficient transfer of authority to local levels, a lack of clarity about the roles and responsibilities of the boards or committees, politicization of the committees’ mandate and the perceptions of community interference in health facility matters which may lead to poor working relationships.^19,66,69^ Molyneux et al indicate that the involvement of health facility boards and community members in facility operations may be viewed as interference by the staff and as undermining their autonomy.^68^

One justification for limiting autonomy and decision space by implementing strict accountability mechanisms is that it prevents corruption. However, the restrictions put in place can exceed what is necessary to achieve this objective and prevent optimal functioning.^45,62^

Increased bureaucratic accountability suggests a stronger monitoring and oversight role for central governments but could reduce the decision space available for local managers. In Uganda and Tanzania, Blas found that the increased bureaucratization – which was in response to the need to prevent mismanagement and misappropriation of funds – led to delays in service delivery.^41^

This reinforces the need to align accountability with a degree of decision space that still allows for effective service delivery.

Accountability mechanisms exist not just to police functioning but also as a means of stewardship to encourage cohesive and co-ordinated policies and standards – particularly in a decentralized system.^42^

However, if decentralization policies are introduced without explicit definition of the lines of responsibility and reporting, management is made more challenging and accountability is undermined by the lack of definition about who can exercise the power to make decisions.^41^

If a system has such fragmented levels of authority and unclear definitions of roles and responsibilities, it is at risk of generating internal conflicts and administrative delays, and undermining accountability because there is lack of clarity about who is responsible.^21,23,28,32,33,71,72^ For example, in South Africa, as a result of deconcentration from the National Department of Health to local departments, and devolution from national to local governments, health services were then accountable to both local and national government which led to confusion about priority setting and planning.^20^

Generating effective accountability mechanisms requires that the organizational structure defines clear lines of authority and decision-making responsibilities between institutions and governance levels.^16,66,70^

This was one of the challenges identified by Heywood and Choi in Indonesia: “As the ability to manage a fractured system is impaired, the other major casualty is that accountability is lost. Eventually no one is held accountable for the performance of the sector – the district blames the center and the central ministries (and their ministers) are not accountable to district populations.”^21^

### Context

Every organization exists within an environment which influences its functioning and operational governance.^12,25,64,73,74^ The degree to which the environment exerts influence blurs the boundaries of the organization and makes successful achievement of objectives dependent on the context-organization relationship.^75^ How the organizational boundary is defined thus affects the decision-making capacity of the organization.

According to Khaleghian, “socio-cultural and political factors can influence the degree of “decision space” provided to local governments, the nature and content of interactions between central and local authorities, the space for local voice in political life, the style of relationships between public officials and community representatives.”^3^ In other words, decision space is not just influenced through vertical interactions from central to decentralized parties but is also influenced horizontally by other actors and organizations operating locally.^73^

The influence of context can be assumed to be greater in federal systems where there are increased lines of convergence between multiple authorities and a greater number of political systems that intersect. Actors then react to changes within the environment they operate in, in accordance with the idea of what acceptable behavior is – whether it be support for a programme that has nationwide consensus, or engaging in corrupt practices because such action is not punished.^76^

Decision space, and in turn priority setting, is significantly influenced by the social and political environment.^30,34,77^ According to Peckham, “A local organization – in this case the health centres – has its autonomy and capacity to act constrained not just by whether it can make autonomous decisions about finance, resource allocation, access, governance etc, it may also be constrained by what is possible in their specific local context.”^78^

The social and political environment also includes the opportunities which actors have to influence policies and implementation as a result of political leadership, socio-economic conditions or public opinion. There may be increased pressure from politicians to fulfill a personal agenda or the public pressure placed on politicians may influence policy priorities or resource allocations.^34,47,79^

“A lack of consideration of the context into which an intervention is introduced can minimize its effectiveness.”^44^ The local context may influence the decision space available as well as the mechanisms through which the benefits of decision space – increased responsiveness, accountability and quality of care – may be realized.^78^ Therefore, it is important to evaluate the context in which the organization exists and identify the features of the environment that influence the way in which the organization functions; the way decisions are made; and potentially, the way in which services are offered.^12^

## Discussion

Aligned with the arguments posited in support of decentralization, the literature suggests that the benefit of increased decision space is the increased opportunity for timely, locally relevant responses. Increased decision space could reduce the bureaucracy surrounding decision-making in the health sector.^7,11^

Our understanding of how decision space functions still needs development and in order to understand how to harness the benefits of decision space, we need to better understand the functions that influence and define it, and the relation dynamics between those functions.

Bossert and Mitchell’s framework presents one conceptualization of how decision space is defined through the interaction with accountability mechanisms and organizational capacity. The benefit of this approach is that it describes the reality of a complex system with interrelated components. The framework suggests that none of the three components yield tangible outcomes by themselves but rather need to influence or be influenced by the other two in order for the system to achieve its objectives. Organizational capacities and accountability mechanisms determine how decisions can be made and which decisions are made.^9^

Based on the literature reviewed, a very rudimentary set of themes on the influence of organizational capacity and accountability mechanisms on decision space can be formed. Of importance in formulating the themes addressed below is the understanding that there are different forms of organizational capacity and accountability mechanisms that may have different influencing effects.

### Accountability Mechanisms and Decision Space

Accountability mechanisms are important for ensuring that decisions are responsive to local needs but also that power is not being abused.^65^ External and bureaucratic accountability mechanisms address these requirements respectively.^66^

The literature suggests that external mechanisms – such as hospital boards – may impact on service providers’ perceived autonomy^68^ but there does not appear to be a direct influence on the functioning of decision space. If anything, if external accountability mechanisms are implemented successfully, if there is greater clarity about the roles and responsibilities required and greater oversight is transferred from the centre to local actors, the available management decision space could be made more responsive to local needs by reinforcing local feedback loops.^19,66,69^

On the other hand, bureaucratic accountability mechanisms that are primarily implemented to curb corruption and mismanagement appear to have a direct influence on the functioning of decision space since they limit the decisions that can be made by local managers.^41,62^

Increased decision space, as a result of the decentralization of responsibilities, necessitates a reduced role for central authorities and thus, reduced bureaucratic control. With a change in the locus of responsibility, comes a change in the level of bureaucratic accountability resulting from a segregation of duties and reduced oversight over all decisions. So, the intention to increase decision space should, theoretically be accompanied by a reduction in bureaucratic accountability.

However, this is not always the case because despite the best intentions to decentralize, central authorities are sometimes reluctant to relinquish power over decision-making, there may be confusion regarding implementation and the lines of responsibility, and the local authorities may not be capacitated to assume the new responsibility.^16,66,70^

### Organizational Capacity and Decision Space

For an organization to function, it requires that the organization be capacitated in terms of financial and human resources as well as management capacity. However, the relationships between each of these capacities and decision space are unclear.

Increased decision space can theoretically yield benefits for the management of human resources^7,34^ but there does not appear to be a direct relationship between increased human resource capacity and increased decision space emerging from the literature reviewed.

Increased decentralized decision space can also have benefits for financial resource management.^11,21,35,38,40-42^ Having increased decision space over the source of finances, funding flows and the budget allows allocations to be based on need, and having greater local control means decisions can be made timeously, reducing administrative delays.^30,38,44^

However it is uncertain what the influence of changes in financial resource capacity may have on decision space. Financial resource capacity can be increased without the decision space being increased or decision space and management responsibility can be increased without being matched by the capacitating resources.^11,34^

If the theoretical benefits of decentralized decision-making are to be realized then decision space should be increased, but it is unclear from the evidence whether financial resource capacities absolutely have to be increased in order to achieve this.

There is limited evidence that suggests that where resource capacities fall short of what is required for organizational functioning, managers may resort to informal decision-making strategies in order to fulfill their responsibilities and mitigate bureaucratic constraints – choosing to stretch the boundaries of the formal decision space granted to them.^44,7,10^

Being able to take advantage of informal decision spaces requires a certain degree of management capacity^10^ and this is one function that does seem to have direct bearing on decision space. To make effective use of increased decision space requires that managers have the capacity, knowledge and skill to implement organizational plans and activities.^30,36,46,53^ In other words, increased decision space requires increased management capacities.

Yet, if managers do not have the resource capacities to ensure organizational functioning, even the most extensive management capacities could be made redundant. Thus, it is important that an organization to which new responsibilities have been ceded, is capacitated in terms of resource, management capacity and decision-making power to meet their organizational objectives.

### Context and Decision Space

These relationships and processes all exist within a specific context, which influences the manner in which they function.^12^ It is challenging to measure the influence of context on decision space because causality cannot be defined within a myriad of potential factors but it is still vital to consider the influence of the social and political features of the system^13^ and to acknowledge their complementarity to Bossert and Mitchell’s framework.

## Conclusion

This review identified literature that supports the idea that organizational capacity and accountability, as well as clarity regarding organizational structure and roles and responsibilities are important for defining the available decision space and their interaction is likely to have an impact on better health system performance.^12^

Importantly, for the benefits of decentralization to be achieved, policies need to address not just *de jure* legislation but should also include implementation plans that encourage *de facto* functioning whereby local managers are enabled to exercise their decision space.^6,11,26^

It has been hypothesized based on the evidence that increased decision space is the result of decreased bureaucratic accountability mechanisms and increased management capacity and that the interaction among these components has the potential to influence system functioning. However, the exact relationship between financial and human resource capacities and decision space still requires extensive investigation. The role of context on system functionality has been acknowledged^12^ but involves too many determinants and causal networks to define in any detail.

The literature reviewed seems to support the position that wider decision space should be accompanied by adequate organizational capacities and appropriate accountability mechanisms.^6,9^ Yet few articles explicitly and substantively addressed the decision space framework’s interdependent relationship dynamics, thus the exploration of these dynamics presented in this review was compiled by synthesizing relevant contributions from various studies. Without further investigation into the causal relationships or associations between decision space, organizational capacity and accountability, as well as health system performance, these findings should be interpreted with caution.

According to Bossert, “the decision space approach attempts to evaluate the effectiveness of different decision space configurations and to provide recommendations to design decentralization processes that will result in better health system performance.”^15^ Organizational management is an important component of health system governance and the decision space framework offers a dynamic approach to assessing the functioning of a complex and interdependent system.

Improving our understanding of the vital system components of decision space, organizational capacity and accountability mechanisms has the potential to improve policy implementation and make improved organizational functioning more attainable. This review intends to contribute to such understanding.

## Acknowledgements

The financial assistance of the National Research Foundation (NRF), Pretoria, South Africa towards this research is hereby acknowledged. Opinions expressed and conclusions arrived at, are those of the author and are not necessarily to be attributed to the NRF.

## Ethical issues

Not applicable.

## Competing interests

Authors declare that they have no competing interests.

## Authors’ contributions

TER conducted the literature search, reviewed the articles, and drafted the manuscript; SC and DM contributed to the conceptualization and towards the writing of the final manuscript.
